# Supplementary material for: Improvements in health-related quality of life are maintained long-term in patients prescribed medicinal cannabis in Australia: The QUEST Initiative 12-month follow-up observational study
Source: PLoS One. 2025 Apr 2;20(4):e0320756. doi: 10.1371/journal.pone.0320756 (PMC11964238; doi:10.1371/journal.pone.0320756)
Supplement: S1 Table — (PDF) [file pone.0320756.s001.pdf]

S1 Table. Condition-specific outcomes assessed including characteristics, scoring, and details of use, for PROMs administered to QUEST participants with diagnosed chronic pain or movement disorder.

Outcome	PROM	Number of items	Description	Rating	Recall period	Scoring	Details
**Pain**							
	PROMIS Scale v2.0 - Pain intensity 3a	3	How much pain hurts at its worse, on average, and right now.	Items rated 1 (no pain) to 5 (very severe).	Past 7 days and now	PROMIS pain measure generates a T-score with a mean of 50 and standard deviation of 10 in a reference population of the US general population (US 2000 Census).[1] Higher scores reflect greater pain intensity.‡	PROMIS Pain intensity has been validated and used extensively in studies of inflammatory arthritis.[2]
	PROMIS Short Form v1.0 - Pain Interference 8a	8	The extent to which pain hinders sleep, enjoyment in life, and engagement with social, cognitive, emotional, physical, and recreational activities.	Items rated 1 (not at all) to 5 (very much so).	Past 7 days	PROMIS pain measure generates a T-score with a mean of 50 and standard deviation of 10 in a reference population of the US general population (US 2000 Census).[1] Higher scores reflect greater pain interference.‡	PROMIS Pain interference has been validated and used in studies of inflammatory arthritis and chronic back pain.[2, 3]
**Motor function**							
	Neuro-QoL Adult Upper Extremity Function – Fine Motor, Activities of Daily Living – Short Form v1.0	8	The ability to perform activities involving digital, manual, and reach-related functions, including self-care.	Items rated 1 (unable to do) to 5 (without any difficulty).	now	Neuro-QoL measures generate a T-score with a mean of 50 and standard deviation of 10 in a reference general population.[1] Higher scores indicate better functioning.‡	Neuro-QoL Upper Extremity Function is a validated measure,[4] used to detect change in patients with stroke, multiple sclerosis, amyotrophic lateral sclerosis, Parkinson disease, epilepsy, and muscular dystrophy.[5]

Neuro-QoL Quality of Life in Neurological Disorders; PROM patient-reported outcome measure; PROMIS Patient-Reported Outcomes Measurement Information System
‡The HealthMeasures Scoring Service recommended for NeuroQoL and PROMIS instruments was used to calculate T-scores using item level calibrations more accurately than manually transforming total raw scores.[6]
All other PROMs administered to QUEST participants are described in detail elsewhere.[7]

References

1. HealthMeasures. PROMIS® Reference Populations 2022 [Dec 2022]. Available from: https://staging.healthmeasures.net/score-and-interpret/interpret-scores/promis/reference-populations.
2. Teuwen MMH, Knaapen IRE, Vliet Vlieland TPM, Schoones JW, van den Ende CHM, van Weely SFE, et al. The use of PROMIS measures in clinical studies in patients with inflammatory arthritis: a systematic review. Qual Life Res. 2023. doi: 10.1007/s11136-023-03422-0.
3. Chen CX, Kroenke K, Stump T, Kean J, Krebs EE, Bair MJ, et al. Comparative Responsiveness of the PROMIS Pain Interference Short Forms With Legacy Pain Measures: Results From Three Randomized Clinical Trials. The journal of pain : official journal of the American Pain Society. 2019;20(6):664-75. doi: 10.1016/j.jpain.2018.11.010.
4. Cella D, Lai JS, Nowinski CJ, Victorson D, Peterman A, Miller D, et al. Neuro-QOL: brief measures of health-related quality of life for clinical research in neurology. Neurology. 2012;78(23):1860-7. doi: 10.1212/WNL.0b013e318258f744.
5. Kozlowski AJ, Cella D, Nitsch KP, Heinemann AW. Evaluating Individual Change With the Quality of Life in Neurological Disorders (Neuro-QoL) Short Forms. Archives of physical medicine and rehabilitation. 2016;97(4):650-4.e8. doi: 10.1016/j.apmr.2015.12.010.
6. PROMIS. PROMIS Sleep Disturbance Scoring Manual 2021 accessed Jul 2022. Available from: https://www.healthmeasures.net/images/PROMIS/manuals/PROMIS_Sleep_Disturbance_Scoring_Manual.pdf.
7. Tait M-A, Costa DSJ, Campbell R, Norman R, Warne LN, Schug S, et al. Health-related quality of life in patients accessing medicinal cannabis in Australia: The QUEST initiative results of a 3-month follow-up observational study. PLOS ONE. 2023;18(9):e0290549. doi: 10.1371/journal.pone.0290549.
