# Supplementary material for: Improvements in health-related quality of life are maintained long-term in patients prescribed medicinal cannabis in Australia: The QUEST Initiative 12-month follow-up observational study
Source: PLoS One. 2025 Apr 2;20(4):e0320756. doi: 10.1371/journal.pone.0320756 (PMC11964238; doi:10.1371/journal.pone.0320756)
Supplement: S2 Table — (PDF) [file pone.0320756.s002.pdf]

The QUEST Initiative 12-month observational study results of HRQL in medicinal cannabis patients

S2 Table. Patient-reported gender identity and ethnicity of 2744 participants recruited to the QUEST Initiative by those included in the 12-month analyses and those who completed baseline only.

Participant Characteristics (n=2744)	Completed baseline only (n=391)	Completed baseline + follow-up (n=2353)	P value (X^2)
Gender identity, n (%)			
Man	142 (36.3)	836 (35.5)	0.857
Woman	224 (57.3)	1404 (59.7)	
Transgender	1 (0.3)	2 (0.1)	
Genderqueer	-	4 (0.2)	
Agender	-	2 (0.1)	
Genderless	-	1 (0.0)	
Non-binary	4 (1.0)	13 (0.6)	
Trans Man	-	1 (0.0)	
Two-Spirit	-	1 (0.0)	
Genderfluid	1 (0.3)	4 (0.2)	
Undisclosed	19 (4.9)	85 (3.7)	
Ethnicity, n (%)			
Aboriginal or Torres Strait Islander	14 (3.6)	54 (2.3)	0.145
Other Australian	216 (55.2)	1309 (55.6)	
New Zealander or Polynesian	6 (1.3)	71 (3.0)	
British	52 (13.3)	374 (15.9)	
Irish	9 (2.3)	71 (3.0)	
North-West European	19 (4.9)	119 (5.1)	
Southern and Eastern European	17 (4.3)	125 (5.3)	
North African and Middle Eastern	6 (1.5)	18 (0.8)	
South-East Asian	4 (1.0)	20 (0.9)	
North-East Asian	4 (1.0)	21 (0.9)	
Southern or Central Asian	7 (1.8)	24 (1.0)	
North American	8 (2.0)	35 (1.5)	
South or Central American	5 (1.3)	31 (1.3)	
Sub-Saharan African	9 (2.3)	24 (1.0)	
Undisclosed	15 (3.8)	57 (2.4)	
