# Supplementary material for: Improvements in health-related quality of life are maintained long-term in patients prescribed medicinal cannabis in Australia: The QUEST Initiative 12-month follow-up observational study
Source: PLoS One. 2025 Apr 2;20(4):e0320756. doi: 10.1371/journal.pone.0320756 (PMC11964238; doi:10.1371/journal.pone.0320756)
Supplement: S3 Table — (PDF) [file pone.0320756.s003.pdf]

The QUEST Initiative 12-month observational study results of HRQL in medicinal cannabis patients

S3 Table. Conditions treated with medicinal cannabis for 2744 participants recruited to the QUEST 12-month study.

**Conditions treated with MC**	**Completed baseline only (n=391)**	**Included in analysis (n=2353)**	**P value (X^2)**
(clinicians selected up to two conditions per patient)**,**	**n (%)**		0.074
Pain - musculoskeletal	140 (35.8)	896 (38.1)	
Pain – neuropathic	79 (20.2)	547 (23.2)	
Sleep disorder	99 (25.3)	546 (23.2)	
Generalised anxiety disorder	90 (23)	520 (22.1)	
Mixed depressive and anxiety disorder	53 (13.6)	263 (11.2)	
Pain - widespread	24 (6.1)	212 (9.0)	
Pain - headache or orofacial	15 (3.8)	128 (5.4)	
Post-traumatic stress disorder	19 (4.9)	127 (5.4)	
Pain – visceral	16 (4.1)	114 (4.8)	
Movement disorder (any)[a]	10 (2.6)	60 (2.5)	
Pain - Cancer related	9 (2.3)	28 (1.2)	
Recurrent depressive disorder	2 (0.5)	25 (1.1)	
Epilepsy	3 (0.8)	18 (0.8)	
Bipolar disorder	0	12 (0.5)	
Pain - Chronic postsurgical or post traumatic	3 (0.8)	10 (0.4)	
Other[b]	22 (5.6)	71 (3.0)	

[a]Movement disorders included: Parkinsonism, tremor, paroxysmal dyskinesias, dystonia, ataxia, and tic disorders.
[b]Other conditions included: attention deficit hyperactivity disorder, autism spectrum disorder, anorexia, bladder irritation with dysuria, bloating, cachexia and wasting, chronic fatigue, chronic gum disease, Congenital Adrenal Hyperplasia, dysmenorrhea, eosinophilic gastroenteritis, Hashimoto's thyroiditis, Irritable bladder syndrome, irritable bowel syndrome, inflammatory bowel diseases, Mast cell activation, nausea and vomiting, Nystagmus, Obsessive compulsive disorder, Perimenopause, Peripheral Neuropathy, Polymyalgia rheumatica, premenstrual dysphoric disorder, Severe eczema, topical steroid withdrawal syndrome.
